# Stenting as a bridge to surgery for extra-colonic malignancy induced colorectal obstruction: preliminary experience

**DOI:** 10.1186/s12876-020-01273-4

**Published:** 2020-04-19

**Authors:** Eui Joo Kim, Sang Hoon Han, Kyoung Oh Kim, Jun-Won Chung, Dong Kyun Park, Kwang An Kwon, Jung Ho Kim

**Affiliations:** 1 Division of Gastroenterology, Department of Internal Medicine, Gachon University Gil Medical Center, Gachon University College of Medicine, 21, Namdong-daero 774 beon-gil, Namdong-gu, Incheon, 21565 Republic of Korea; 2 Department of Internal Medicine, Jeju National University Hospital, Jeju National University School of Medicine, Jeju, Republic of Korea

**Keywords:** Colonic obstruction, Bridge to surgery, Extra-colonic malignancy, Endoscopy, Self-expanding metal stent

## Abstract

**Background:**

The majority of colonic obstructions result from colorectal cancer. However, malignancies of extra-colonic origin can also disrupt colorectal patency, and the efficacy of self-expanding metal stents (SEMS) insertion as a bridge to surgery in these patients are still in debate. The aim of this study is to evaluate the efficacy of endoscopic stenting as a bridge to surgery (BTS) for extra-colonic malignancy (ECM)-induced colonic obstruction.

**Methods:**

Thirty-three patients with colonic obstruction due to ECM who received self-expanding metal stents (SEMS) insertion at a single academic tertiary medical center between 2004 and 2015 were included. The purpose of SEMS insertion was determined based on whether the patient’s medical records indicated any surgical plans before SEMS insertion. Technical success was defined as a patent SEMS covering the entire length of the obstruction. Bridging success was defined as elective surgical procedures after the first SEMS insertion.

**Results:**

Among the 33 patients who underwent SEMS insertion for colorectal obstruction due to ECM, nine underwent SEMS as a BTS. Technical success was achieved in 100% (9/9). Seven patients underwent elective surgery after successful decompression with the first SEMS, and the bridging success rate was 77.8% (7/9). Two patients needed secondary stent insertion before elective surgery. However, none of them required emergent surgery. No major complications occurred, including death related to colorectal endoscopic procedures, perforation, or bleeding.

**Conclusion:**

SEMS insertion as a BTS is a good treatment option to avoid emergent surgery in patients with colonic obstruction caused by extra-colonic malignancy.

## Background

Since the early 1990s, self-expanding metal stents (SEMSs) have been used for palliation of colorectal obstruction caused by inoperable gastrointestinal malignancies. Owing to the continuous evolution of the SEMS over > 20 years, the clinical indication of SEMS insertion in colorectal obstruction has expanded beyond palliative purposes, and SEMS insertion as a bridge to surgery (BTS) may be a feasible clinical choice [1, 2].

The pathophysiological mechanisms of obstruction by extra-colonic malignancy (ECM) include extrinsic compression, intramural compression, mesenteric infiltration, and dysmotility [3–5]. The mechanism of colorectal obstruction due to ECM would theoretically differ from that of intrinsic luminal obstruction caused by luminal space–occupying primary colorectal cancer, so the efficacy of SEMS insertion is expected to differ in colorectal obstruction due to ECM.

Some reports have attempted to elucidate the efficacy and success rate of SEMS insertion in cases of colorectal obstruction due to ECM [6–8]. However, no studies have reported the clinical efficacy of using colonic stents as a BTS in patients with colorectal obstruction caused by ECM because of the low incidence and heterogeneous characteristics of colorectal obstruction caused by ECM. Therefore, consensus is lacking for the SEMS insertion as a BTS for colorectal obstruction caused by ECM.

The expected clinical effect of using SEMS differs depending on whether the purpose is a palliation or a BTS. Although palliation should maintain a function during long-term survival, the bridge should be effective until the patient’s condition stabilizes and the elective planned operation is successful in one stage. In other words, when a SEMS is used as a BTS, the goal is the achievement of a successful surgical outcome without complications [9, 10]. However, to our knowledge, no report has described its clinical efficacy, including surgical outcomes after stenting as a BTS in patients with ECM-induced colorectal obstruction.

Therefore, in the present study, we aimed to determine the clinical efficacy and surgical outcomes of SEMS placement as a BTS in patients with colonic obstruction due to ECM.

## Methods

### Patients

The medical records of patients who underwent SEMS placement for a colorectal obstruction at a single academic tertiary medical center between July 2004 and December 2015 were retrospectively reviewed. The inclusion criteria were SEMS insertion as a BTS, non-colorectal cancer, obstructive symptoms and/or signs, and colonoscopic and radiological findings of colorectal obstruction. Patients with SEMS insertion for palliation, primary colorectal cancer, a history of colorectal resection, metastatic colorectal cancer, and benign stricture were excluded.

All the patients had large bowel obstructions caused by non-colorectal malignancies and had no history of previous SEMS placement. All the patients exhibited clinical features of colorectal obstruction, such as abdominal pain, abdominal distension, constipation, nausea, and vomiting. Plain abdominal radiographs showed dilated colons, and colonic obstruction was confirmed by using computed tomography or colonoscopy with fluoroscopy prior to SEMS deployment. The purpose of SEMS insertion as BTS was determined on the basis of whether the patient’s medical records indicated any surgical plans before SEMS insertion.

**Fig. 1:**
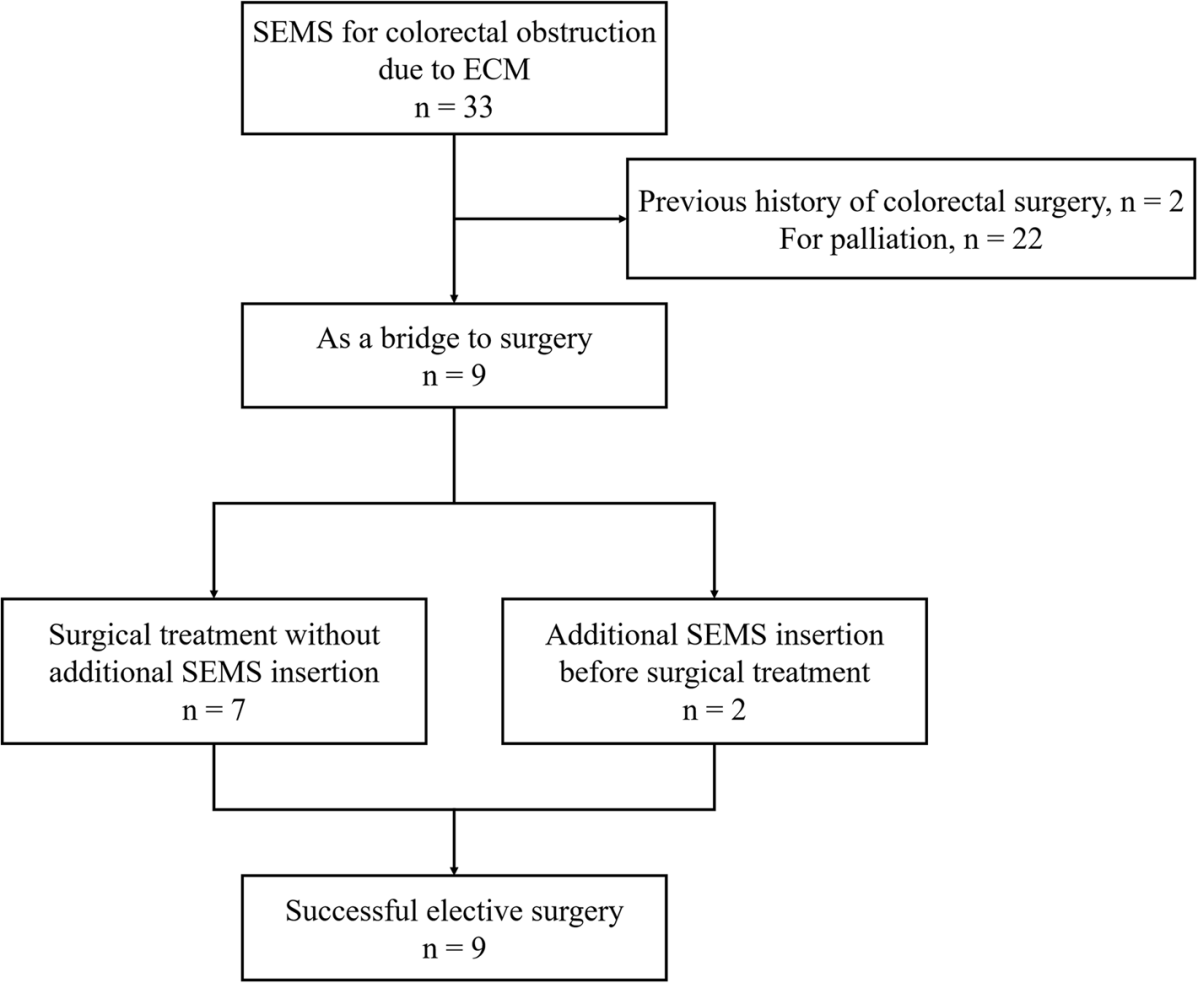
Flowchart of the study. SEMS, self-expanding metal stent; ECM, extra-colonic malignancy

Thirty-three patients with colonic obstruction due to ECM were treated with SEMS insertion during the study period. Two patients with a history of previous colonic surgery that might change the normal anatomy of the colon and 22 patients who had an SEMS insertion for palliation were excluded. Nine consecutive patients who underwent SEMS placement as a BTS for colorectal obstruction due to ECM were finally included in our study (Fig. 1). This study was approved by the Institutional Review Board/Ethics Committee (IRB no. GDIRB2016–071) and formal consent is not required for this type of study.

### Procedures

In all the patients, SEMS was inserted to decompress the colorectal obstruction as a BTS. SEMS insertion was performed under simultaneous endoscopic and fluoroscopic guidance [4, 10, 11]. Informed consent was obtained from each patient after an explanation of the procedure, and possible complications were given. GIF-2 T240 (Olympus Optical Corp, Tokyo, Japan) and CF-Q240L (Olympus) were used for all of the procedures. Any one of ComVi Enteral Colonic Stent, Niti-S stent (Taewoong Medical Co., Seoul, Korea), and Hanarostent (M. I. Tech, Seoul, Korea), stents commercially available in the Republic of Korea, was used. All the patients received an enema with or without intramuscular pethidine (25 mg) before the procedure.

After the obstructive lesion was identified on endoscopy, a contrast medium was injected and the morphology and length of the obstructive lesion were assessed on the basis of previously acquired computed tomography and fluoroscopy. After the stent type, size, and length were determined by experienced endoscopists who had at least 5 years of therapeutic endoscopic experience, a guidewire was introduced through the obstructive lesion under endoscopic and fluoroscopic guidance. Once the guidewire was passed through the lesion of interest, the metal stent delivery catheter was advanced over the guidewire through the endoscope until the stent was positioned against the obstructive lesion. Upon releasing the stent delivery catheter, the SEMS was deployed progressing distally. Proper SEMS expansion and position were assessed by a series of plain abdominal radiographs taken during the hospitalization.

After the successful decompression with SEMS insertion as a BTS, a preoperative evaluation was performed and elective surgery was planned by experienced surgeons specializing in coloproctology, in accordance with the assessment of the patient’s bowel function and clinical condition. Patients with successful decompression with SEMS insertion received bowel preparation with 2–4 l of polyethylene glycol 1 day before the planned surgery. The surgeon determined the type of surgery depending on the location of the primary disease and the intraoperative conditions of the patient. Primary tumor resection and anastomosis were planned and performed as possible.

### Definition

Technical success was defined as a patent SEMS covering the entire length of the obstruction. Bridging success was defined as elective surgical procedures after the first SEMS insertion because SEMS deployment in the BTS group is performed to create temporary decompression as a preoperational preparation. Surgical failure was thus inclusive of technical failure, insufficient decompression before the planned surgery, and unplanned emergent surgery. Unlike previous studies that aimed to determine the surgical outcome of SEMS insertion as a BTS, we did not consider primary anastomosis as an endpoint. In patients with ECM, primary anastomosis might be the preferable surgical option because considering the stage of primary malignancy, recurrent obstruction of other sites after surgery can be expected.

### Statistical analysis

Statistical analysis was performed using SPSS for Windows version 23.0 (IBM Corporation, Armonk, NY, USA). Continuous variables are presented as mean (SD) values; and categorical variables, as numbers and percentages.

## Results

### Baseline characteristics

The mean age of the included patients was 58.9 (19.9) years. Among the nine patients, the most common cause and site of colonic obstruction were gastric cancer (44.4%) and, splenic flexure (33.3%), respectively. Of the patients, five (55.6%) had a previous history of surgery in the abdominal cavity that did not involve the colon, and two (22.2%) exhibited peritoneal carcinomatosis on enhanced abdominal computed tomography at the time of SEMS insertion (Table 1). Eleven SEMSs were used preoperatively in nine patients. For the 11 SEMSs, the most common lengths were 80 (44.4%) and 100 mm (44.4%), and the most common diameter was 24 mm (54.5%). Of these stents, eight (72.7%) were uncovered, two (18.1%) were partially covered, and only one (9.1%) was covered (Table 2).

**Table 1:** Clinical characteristics of patients

Case No	Sex	Age (range)^a^	Cancer origin	Obstruction site	PC	Multi-site obstruction	Previous treatment before colonic obstruction	Neoadjuvant systemic chemotherapy	FUD (d)	Expire
1	F	50–55	Cervical cancer	Upper rectum	No	No		No	109	Yes
2	F	90–95	Pancreas cancer	Splenic flexure	No	No		No	186	Yes
3	M	60–65	AGC	Transverse	Yes	No	Palliative radical total gastrectomy with chemotherapy	No	85	No
4	F	40–45	MUO	Sigmoid	No	No		No	60	No
5	M	55–60	Pancreas cancer	Sigmoid	Yes	No	Distal pancreatectomy	No	312	Yes
6	M	80–85	AGC, RCC	Descending	Yes	No	Nephrectomy Radical total gastrectomy	No	42	No
7	M	55–60	AGC	Splenic flexure	Yes	No	Radical total gastrectomy	No	288	No
8	M	50–55	Pancreas cancer	Splenic flexure	Yes	No		Yes^b^	287	Yes
9	F	25–30	AGC	Descending	Yes	No		Yes^c^	463	No

*F* female; *M* male; *PC* carcinomatosis peritonei; *FUD* follow up duration; *AGC* advanced gastric cancer; *MUO* Malignancy of undefined primary origin; *RCC* renal cell carcinoma

^a^Age was expressed in a range to maintain anonymity

^b^FOLFIRINOX

^c^Trastuzumab, capecitabine and cisplatin

**Table 2:** Clinical outcomes of SEMS insertion as BTS

Case no.	Stent characteristics			Technical Success	Bridging Success	Cause of failure	Follow-up treatment	ISS (days)	OP Name	ASOS
	SEMS type	Length (mm)	Diameter (mm)							
1	Partially covered	120	20	Yes	No	Migration	2nd Uncovered SEMS before surgery	12	Loop ileostomy	
2	Uncovered	100	24	Yes	Yes		Surgery	5	Left hemicolectomy, Wedge resection of pancreas body	
3	Uncovered	80	20	Yes	Yes		Surgery	8	Ileostomy	
4	Uncovered	100	24	Yes	No	Insufficient expansion	2nd partially covered SEMS before surgery	15	LAR, Salphingoophorectomy	
5	Covered	80	20	Yes	Yes		Surgery	32	Segmental resection and anastomosis of S-colon	Yes
6	Uncovered	100	24	Yes	Yes		Surgery	33	Lap-loop ileostomy, Palliative	
7	Uncovered	100	24	Yes	Yes		Surgery	16	Cecum-Sigmoid Colon bypass	
8	Uncovered	80	24	Yes	Yes		Surgery	64	Open and closure	Yes
9	Uncovered	80	24	Yes	Yes		Surgery	357	TG, BSO, T-colon segmental resection	

*SEMS* self-expanding metal stent; *BTS* bridge to surgery; *ISS* interval from stent insertion to surgery; *OP* operation; *ASOS* another site obstruction after surgery; *F* female; *M* male; *LAR* lower anterior resection; *TG* total gastrectomy; *BSO* bilateral salpingo-oophorectomy

### Success rates and clinical outcomes

The technical success rates in the patients who underwent SEMS insertion as a BTS was 100% (9/9), while the bridging success rate was 77.8% (7/9; Table 2). In one patient (Case 1), stent (partially covered SEMS) migration occurred within 48 h and a secondary SEMS (uncovered SEMS) was inserted before the surgery. Another patient (Case 4) needed a secondary SEMS insertion because although the clinical symptom was improved with the first SEMS insertion, the SEMS expansion was insufficient for the bowel preparation required for the surgery. Abdominal pain developed with bowel preparation, so surgery was performed after successful secondary SEMS insertion.

Although two patients required secondary SEMS insertion, elective surgical treatments were administered successfully without complications (Fig. 1). Four patients underwent successful resection of the obstructive lesion and primary anastomosis, while another three patients with loop ileostomy and one patient underwent cecal-sigmoid colon bypass. Unfortunately, in one patient with pancreatic cancer, because severe peritoneal seeding was noted intraoperatively, no surgical treatment was possible (open and closure). Consequently, none of the patients required emergent decompressive surgery. However, because two patients needed additional management before planned surgery due to insufficient decompression and stent migration, the bridging success rate was 77.8% (7/9; Table 2).

### Complications

Of the nine patients, one (11.1%) experienced stent migration. However, no major complications occurred, including death related to colorectal procedures, perforation, or bleeding. In one patient, surgical resection of the obstructed site was impossible because of severe peritoneal carcinomatosis. During the postoperative follow-up period, after successful segmental resection and anastomosis of the sigmoid colon with SEMS insertion as a BTS, one patient with pancreatic cancer required reintervention with SEMS because of additional colonic obstruction at the site other than the previous anastomosis site. The remaining seven patients did not require additional reintervention during the follow-up period after surgery.

## Discussion

Compared with the incidence of colorectal obstruction caused by primary colorectal cancer, that of colorectal obstruction caused by extra-colonic compression is relatively low (4.7–8.5%) [7, 12, 13]. Although the incidence is higher than expected, reports on the effectiveness and clinical course of SEMS treatment are rare. The present study described a long-term clinical experience to show the effectiveness of SEMS insertion as a BTS in a homogeneous group of patients with ECM-induced colorectal obstructions, which have not been reported previously.

In the present study, nine patients with colonic obstruction due to extra-colonic origin underwent SEMS insertion as a BTS. The overall technical success rate was 100% (9/9) and the bridging success rate was 77.8% (7/9), which are similar to those of colonic stent placement as a BTS (70.7–96.2% and 46.7–100%, respectively) in previous studies that included patients with colorectal obstruction caused by primary colorectal cancer [4, 14–18].

The pathophysiological mechanisms of colorectal obstruction by ECM include extrinsic compression, which is caused by the mass effect of intra-abdominal tumor and adhesions; intramural compression, which may result in poor motility caused by tumor invasion of the bowel wall; mesenteric infiltration, which may change the angulation of the bowel; and tumor infiltration into the enteric or celiac plexus, which causes dysmotility [3, 4]. Despite the different mechanisms, it is interesting that the results are similar to the success rates in patients with primary colon cancer obstruction.

In our study, the SEMS migration rate was 11.1% (1/9), and perforation and bleeding, which are known as major critical complications, did not occur. The complication rates in our study were not as high as those of previous studies that reported stent migration rate of 1.0–12.5%, perforation rates of 0–12.8%, and bleeding rates of 0–3.7% [1, 2]. According to these results, SEMS insertion in colorectal obstruction due to ECM was not only effective for decompression but also showed a good safety profile.

During the postoperative follow-up period, two patients (22.2%) with pancreatic cancer required further intervention. In one patient, additional SEMS insertion was required even after segmental resection and anastomosis during the follow-up period. The other patient needed an additional SEMS insertion during cancer progression because resection of the lesion was not possible because of severe peritoneal carcinomatosis. Pancreatic cancer has fewer chemotherapy options than other gastrointestinal cancers and is known to have a poor prognosis [19]. Considering this fact, for gastrointestinal cancers such as colorectal and gastric cancers, with a wide range of postoperative chemotherapy options, a better prognosis and lower reintervention rate after successful elective surgery can be expected [20, 21].

In most of the cases in our study, uncovered SEMSs were used. Higher migration rates of covered SEMSs were reported in several studies of colorectal malignancy, and because most of the patients in this study did not have definitive luminal masses, even higher migration rates of covered SEMS can be expected [22, 23]. However, evidence is not enough to reach a consensus that supports the idea that uncovered SEMS would show more favorable results than covered SEMS in ECM-induced colorectal obstruction. Owing to the paucity of patients in whom covered SEMS was deployed in this study, further studies are required to resolve this issue.

However, some concerns have been raised about SEMS insertion as a BTS for ECM-induced colorectal obstruction. Most cases of colorectal obstruction due to ECM have peritoneal carcinomatosis or intra-abdominal metastatic mass, which suggests that other multiple obstructive sites may exist. In multiple site obstructions, a single SEMS insertion might not resolve the obstruction before the surgical treatment or re-obstruction at another site may occur.

However, in our study, two patients required additional SEMS insertion before elective surgery because of SEMS expansion insufficiency and stent migration, and none of the patients required additional preoperative SEMS insertion because of another site obstruction causing decompression failure. Although 6 patients presented with carcinomatosis peritonei, none had multiple site obstructions. Probably, this is because this is a retrospective study. In the patients with suspected multiple site obstructions, SEMS insertion might not have been attempted. Still, our data suggest that even with carcinomatosis peritonei, if the culprit obstruction site is a single lesion, SEMS as a BTS is a feasible choice with a good safety profile.

Case 9 in this study is an interesting case in which stenting had an excellent effect on ECM-induced colonic obstruction. The patient was a 28-year-old woman with a very good performance status. As with other patients, she planned to undergo the operation as soon as possible after stenting. However, her symptoms improved much more than expected after the stent procedure, so she wanted to postpone the surgery. In addition, she showed good response to the chemotherapy after stenting. As a result, the interval between the surgery and the stenting was 357 days. In this patient, use of the stent may be mistaken as a palliative therapy rather than as a BTS. However, because this was a retrospective study, maintaining the reliability of the study is important. We used all the intention-to-treat groups for which stenting for BTS was planned. Therefore, to maintain the reliability of the study, the patient’s data were included in the analysis as originally planned.

Our study is limited by its small number of patients, retrospective design, and possible selection bias because SEMS insertion as BTS might not have been tried in patients with suspected multiple colorectal obstructions caused by ECM. However, although colorectal obstruction caused by ECM is rare, because long-term survival of various cancer patients is improving owing to the development of new anticancer treatment modalities, the incidence of colorectal obstruction by ECM might increase in the future. The results of our study can provide fundamental evidence for the future larger case-controlled studies.

## Conclusions

SEMS insertion as a BTS is a good treatment option to avoid emergent decompressive surgery for patients with ECM-induced colorectal obstruction. This result could aid in the establishment of a consensus regarding an updated treatment strategy for these patients.

## Data Availability

The datasets generated or analysed during the current study are available from the corresponding author on reasonable request.

## Abbreviations

AGC: Advanced gastric cancer
BTS: Bridge to surgery
ECM: Extra-colonic malignancy
FU: Follow up duration
MUO: Malignancy of undefined primary origin
PC: Carcinomatosis Peritonei
SEMS: Self-expanding metal stents
RCC: Renal cell carcinoma
